# Microbial Fermentation of Polyethylene Terephthalate (PET) Plastic Waste for the Production of Chemicals or Electricity**

**DOI:** 10.1002/anie.202211057

**Published:** 2022-10-10

**Authors:** Shafeer Kalathil, Melanie Miller, Erwin Reisner

**Affiliations:** ^1^ Yusuf Hamied Department of Chemistry University of Cambridge Cambridge CB2 1EW UK

**Keywords:** Biosynthesis, Fermentation, *Ideonella Sakaiensis*, Electrosyntrophy, Plastic Recycling

## Abstract

*Ideonella sakaiensis* (*I. sakaiensis*) can grow on polyethylene terephthalate (PET) as the major carbon and energy source. Previous work has shown that PET conversion in the presence of oxygen released carbon dioxide and water while yielding adenosine triphosphate (ATP) through oxidative phosphorylation. This study demonstrates that *I. sakaiensis* is a facultative anaerobe that ferments PET to the feedstock chemicals acetate and ethanol in the absence of oxygen. In addition to PET, the pure monomer ethylene glycol (EG), the intermediate product ethanol, and the carbohydrate fermentation test substance maltose can also serve as fermenting substrates. Co‐culturing of *I. sakaiensis* with the electrogenic and acetate‐consuming *Geobacter sulfurreducens* produced electricity from PET or EG. This newly identified plastic fermentation process by *I. sakaiensis* provides thus a novel biosynthetic route to produce high‐value chemicals or electricity from plastic waste streams.

## Graphical abstract

Microbial degradation of poly(ethylene terephthalate) (PET) with *Ideonella sakaiensis* releases the greenhouse gas carbon dioxide. Here, a fermentative metabolic pathway in *Ideonella sakaiensis* was uncovered facilitating PET degradation while producing valuable feedstock chemicals or electricity in a microbial fuel cell with *Geobacter sulfurreducens*. This ‘plastic fermentation’ process provides opportunities for future bioenergy technologies while combating plastic pollution.

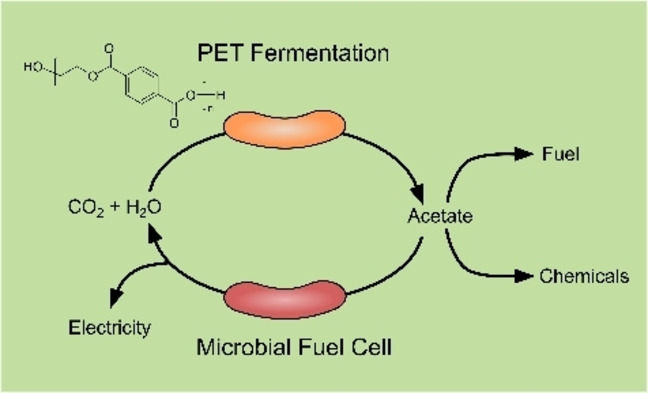

## Introduction

Synthetic organic polymers known as plastics are commonly used in many applications such as construction, the electronics industry, and packaging due to their high durability, low price, easy processability, and low weight, but the majority are discarded after a single use, causing severe environmental concerns.[^1^, ^2^, ^3^] Among them, polyethylene terephthalate (PET) is widely used in drinking bottles, packaging materials, and fibers in the textile industry. The annual total plastic production in 2019 was 368 million tons of which PET‐based plastics contributed approximately 30 million tons.^[4]^ Mechanical recycling of PET plastic is widely used but the process results in a decrease in quality and the low demand for such lower‐quality plastics limits mechanical recycling to a few cycles.[^5^, ^6^, ^7^] Among chemical PET recycling methods are hydrolysis, methanolysis, glycolysis, and aminolysis, which usually require high temperature and can cause environmental pollution.^[8]^ The non‐recycled PET plastics are mostly disposed by landfilling or incineration.^[9]^ Another route for plastics degradation is destruction of larger plastics to micro‐ and nano‐plastics by ultraviolet light exposure together with mechanical disruption.^[10]^ However, micro‐ and nano‐plastic particles are believed to enter the food chain, which causes a serious concern to health.[^11^, ^12^] Thus, a sustainable and green method to mitigate plastic wastes is highly warranted.

Most of the synthetic plastics, including PET, are chemically inert. Nevertheless, several enzymes and microbes have been identified to break down the PET co‐polymer into its corresponding monomers.[^10^, ^13^] Among those enzymes, PETase, carboxylesterase, polyester hydrolase, lipase, and cutinase are known for the degradation of PET at ambient temperature and benign pH.[^8^, ^10^, ^14^, ^15^, ^16^, ^17^, ^18^] Although microbial degradation is currently too slow for commercial applications, improvements in protein/metabolic engineering may ultimately provide a sustainable solution to degrade plastic waste.[^10^, ^13^, ^19^, ^20^, ^21^] Recently, the bacterium *I. sakaiensis* has been identified to degrade and assimilate PET as its sole carbon and energy source under aerobic conditions.[^13^, ^19^, ^20^, ^21^] The bacterium initially adheres on the surface of PET and produces the intermediate mono(2‐hydroxyethyl)‐terephthalic acid (MHET) by secreting extracellular PETases. The MHET is then transported into the periplasm through an outer membrane protein such as porin. MHETase, an intracellular lipoprotein, then hydrolyzes the MHET to terephthalic acid (TPA) and EG. *I. sakaiensis* metabolizes the hydrolyzed products to yield ATP by oxidative phosphorylation via the tricarboxylic acid (TCA) cycle. During this process of cellular respiration in the presence of oxygen, *I. sakaiensis* grows (biomass is produced) and the fully oxidized end products CO_2_ and H_2_O are released.^[19]^ In contrast, in the absence of oxygen, many microorganisms are capable of fermentation, which is generally a slower process that produces less ATP and releases small organic molecules such as lactate, acetate, ethanol, and others as end products.

**Figure 1:**
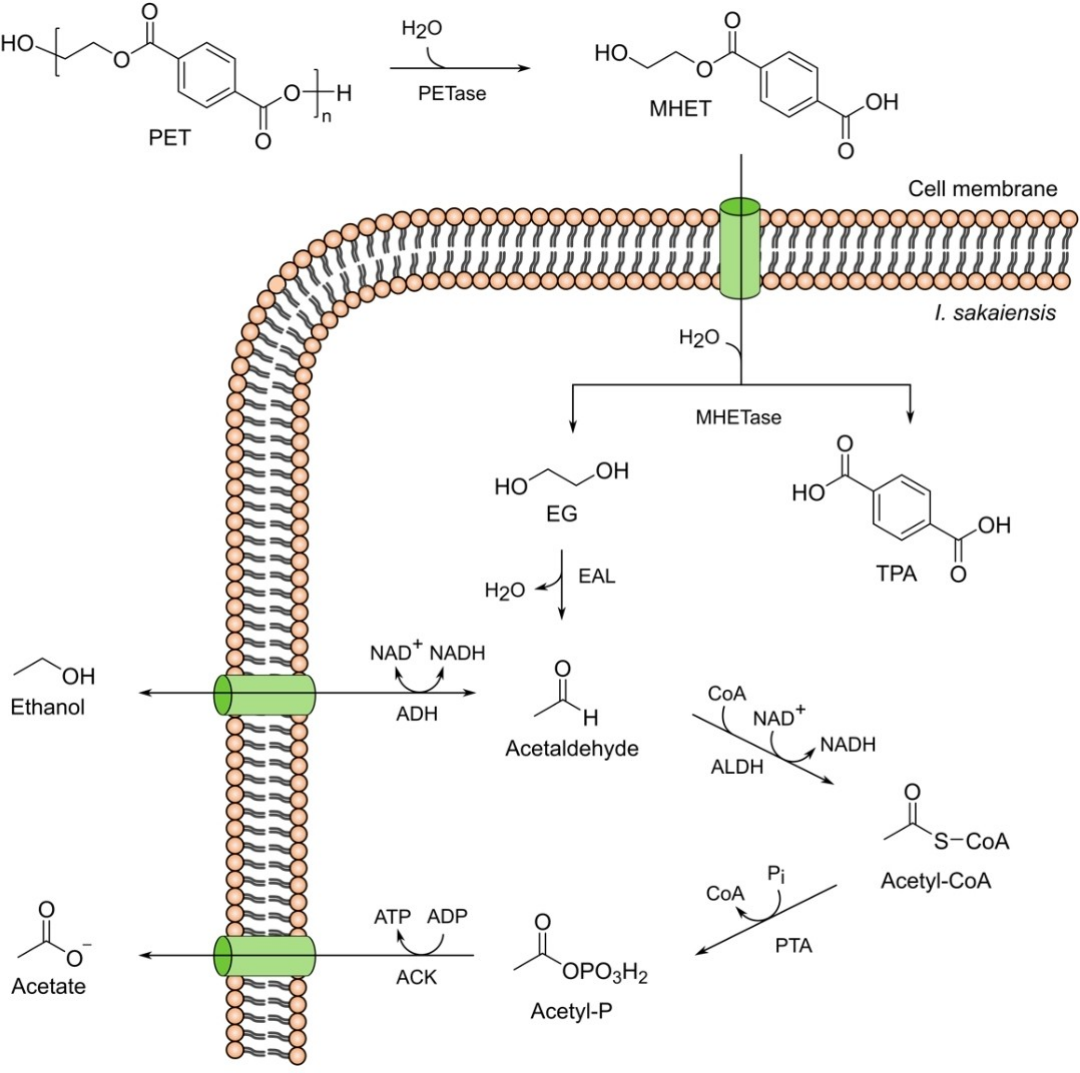
Anaerobic PET conversion by *I. sakaiensis. I. sakaiensis* secretes the enzyme PETase which converts PET into MHET, which is then transported inside *I. sakaiensis* and is hydrolyzed by MHETase to terephthalic acid (TPA) and ethylene glycol (EG). Under anaerobic conditions, we propose that EG is first dehydrated to acetaldehyde by ethanolamine ammonia lyase (EAL) and then converted by alcohol dehydrogenase (ADH) and aldehyde dehydrogenase (ALDH) to ethanol and acetyl‐CoA, respectively. The latter is further converted into acetate by phosphate acetyl transferase (PTA) and acetate kinase (ACK) coupled to ATP formation by substrate‐level phosphorylation. Both, acetate and ethanol are transported outside of the cell. Ethanol can reenter the cell and be converted further into acetate.^[22]^

So far, *I. sakaiensis* has only been considered as an aerobe.[^19^, ^21^] In this study, we show that *I. sakaiensis* is a facultative anaerobe that can ferment PET into acetate and ethanol under anaerobic conditions while yielding ATP by substrate‐level phosphorylation (Figure 1). Fermentation was only observed at high optical densities measured at 600 nm (OD_600_) of approximately 1.2 to 1.4, which is a likely reason why this pathway has previously been overlooked.^[21]^ In addition to PET, other substrates such as maltose, EG, and ethanol can also be fermented into value‐added chemicals by *I. sakaiensis*. Co‐culturing of *I. sakaiensis* with the bacterium *G. sulfurreducens* attached on a porous inverse opal indium tin oxide (IO‐ITO) electrode in a microbial fuel cell produced electricity from PET or EG, where *I. sakaiensis* provided acetate as a substrate from the fermentation of PET or EG for the immobilized *G. sulfurreducens*. Instead of producing H_2_O and the energetically unfavourable greenhouse gas CO_2_ as the end products from PET via cellular respiration, we demonstrate in this work that under fermentation conditions, *I. sakaiensis’* metabolism can be used for the production of high‐value chemicals or, upon co‐culturing with *G. sulfurreducens*, the generation of electricity from PET. Both pathways provide an economic opportunity to mitigate plastic pollution while co‐producing chemicals and energy.

## Results and Discussion

### PET and EG Fermentation by *I. sakaiensis*

*I. sakaiensis* is a gram‐negative, rod‐shaped bacterium with a cream color (Figure S1) after incubation in the culture medium (NBRC no. 802, Table S1) under aerobic conditions for 24 h. *I. sakaiensis* was originally isolated from sediments nearby a plastic recycling plant[^19^, ^21^] and sediments can show frequent shifts between aerobic and anaerobic states.^[23]^ Therefore, microbial communities in sediments often show a facultative trait, which means that they can survive in more than one specific condition and do not only show aerobic respiration but also have the ability to ferment and utilize hydrogen. This is often indicated by a rich abundance of fermenting and hydrogenase enzymes in the genome.^[23]^ Fermentation usually yields organic acids and hydrogenases keep the redox balance by regulating the proton‐hydrogen equilibrium.[^24^, ^25^] Specifically, the genome of *I. sakaiensis* shows the presence of [NiFe] hydrogenases and many fermenting enzymes such as carbon monoxide dehydrogenases, lactate dehydrogenases, alcohol dehydrogenases, acetate kinase among others (Table S2). Additionally, genes predicted to code for proteins involved in the anaerobic metabolism such as iron‐sulfur proteins, cytochrome c family proteins, nitrate reductase, nitrite reductase, sulfite reductase, and dimethyl sulfoxide reductases as well as CRP/FNR transcriptional regulators (anaerobic regulatory proteins) and Fur (a ferric uptake regulator protein) are present in the genome of *I. sakaiensis* (Table S2).

The gene analysis therefore reveals an abundance of fermenting and anaerobic enzymes in the genome of *I. sakaiensis*, which inspired us to investigate its fermentative metabolism. *I. sakaiensis* can grow aerobically with PET as the substrate yielding CO_2_ as the end product,^[19]^ and we demonstrate here the fermentative growth of *I. sakaiensis* with PET as the sole carbon and energy source. The fermentation was carried out with *I. sakaiensis* (OD_600_=1.2–1.4) in 15 mL bicarbonate‐buffered medium (Table S3) in an anaerobic vial with PET films (60 mg) as the sole carbon source inoculated at ambient conditions (pH 7, 30 °C).

**Figure 2:**
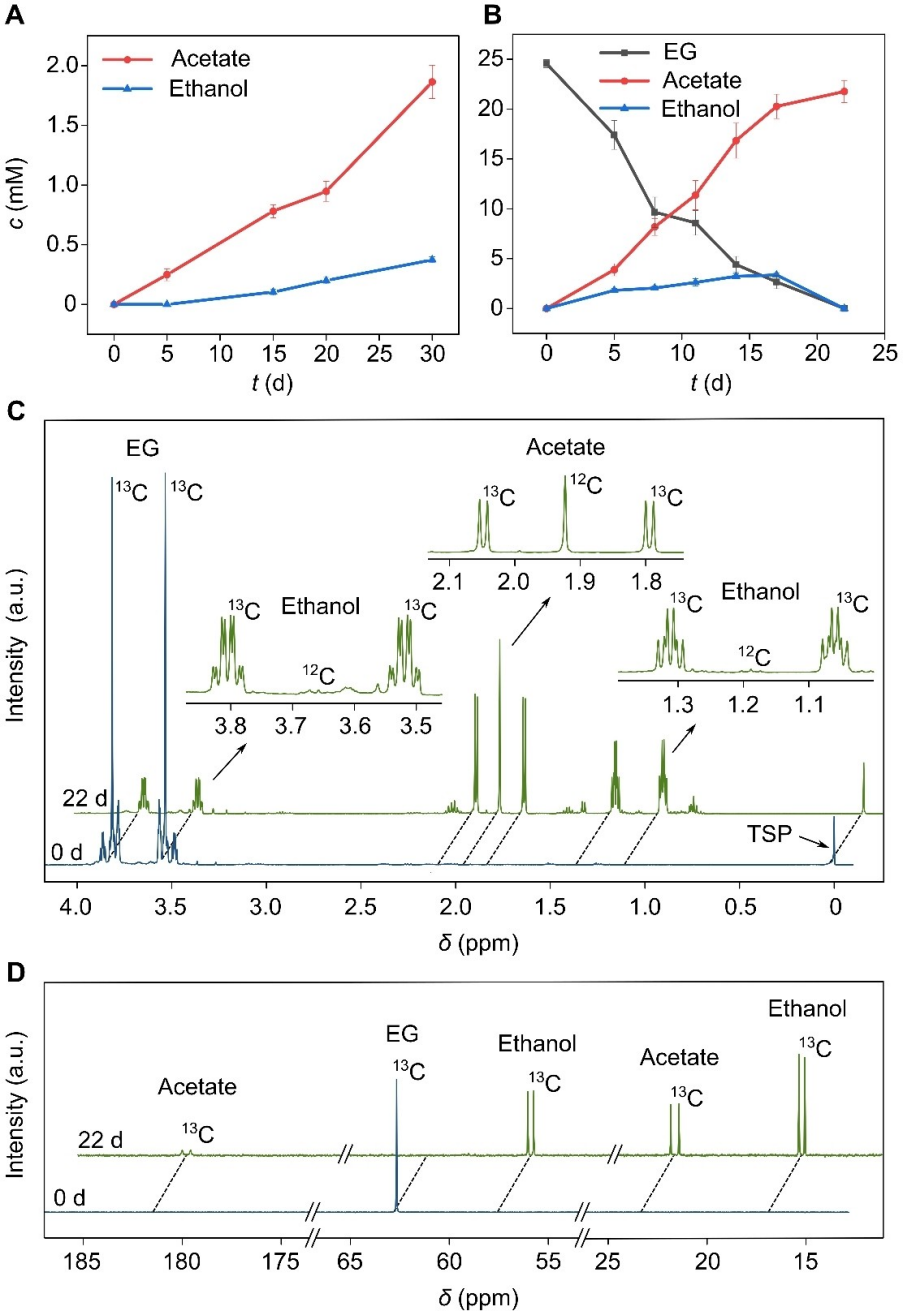
Anaerobic PET and EG fermentation by *I. sakaiensis*. Conditions: A) 60 mg PET film and B) 25 mM EG, *I. sakaiensis* OD_600_=1.2–1.4, 15 mL bicarbonate‐buffered medium, N_2_‐CO_2_ (80–20 %), shaking incubator, 300 rpm, 30 °C, pH 7. Error bars correspond to standard deviation (N=3). C) ^1^H NMR and D) ^13^C NMR spectra showing product formation after 22 d (green) from 25 mM ^13^C labelled EG at 0 d (blue). The TSP signal indicates 0 ppm in each spectrum. The 22 d spectra are vertically and horizontally shifted as indicated by the black dashed lines.

After 30 d of anaerobic incubation, 45 mg of the PET films were consumed (PET degradation yield of 75 %), while 1.9 mM acetate and 0.4 mM ethanol were produced (Figure 2A). ^1^H nuclear magnetic resonance (^1^H NMR) spectroscopy with trimethylsilylpropanoic acid (TSP) as the internal standard was used for the quantification. This corresponds to a PET degradation rate of 7.8 μmol d^−1^ with a product formation rate from PET of 1.2 μmol d^−1^, corresponding to a total PET‐to‐acetate/ethanol conversion yield of approximately 15 mol % (12 mol % acetate and 3 mol % ethanol). After 30 d, it is likely that soluble oligomers are accumulating in solution from PET degradation while monomeric EG is not yet accessible for further conversion into acetate and ethanol by *I. sakaiensis*, explaining the low conversion yield of 15 mol %. No products from TPA degradation have been identified by ^1^H NMR spectroscopy after 30 d (Figure S2). However, as monomeric TPA and EG are only released inside the cells, we cannot exclude intracellular accumulation of TPA or its degradation products. This result suggests hydrolysis of PET into its monomers (TPA and EG) via soluble oligomers, followed by fermentation of EG into acetate and ethanol.

To verify this hypothesis, *I. sakaiensis* was directly grown on EG as the sole substrate. After 22 d, EG (25 mM) was fully consumed, while the acetate concentration constantly increased over time reaching 21.8 mM after 22 d. The only other detectable product was intermittently produced ethanol, which reached its highest concentration of 3.3 mM after 17 d and was then completely depleted after 22 d (Figure 2B and S3). EG degradation occurs at a rate of 17.1 μmol d^−1^ after 22 d with a product formation rate of 14.8 μmol d^−1^. This results in an overall EG‐to‐acetate conversion yield after 22 d of approximately 87 mol % with the remaining amount of EG likely being used for *I. sakaiensis* biomass production. The EG‐to‐acetate conversion yield is significantly higher than the PET‐to‐acetate/ethanol conversion yield, indicating that substrate accessibility is likely limiting in the case of PET.

Control experiments without *I. sakaiensis* and with heat‐killed *I. sakaiensis* cells did not yield any products (Table S4). Experiments in the absence of PET and EG resulted in no ethanol and 0.3 mM acetate production after 30 d, which corresponds to less than 2 % of total acetate produced with EG after 22 d (Table S4). This formation of acetate in the absence of substrate is likely a result of carbon storage compounds inside *I. sakaiensis*. These control experiments confirm that both, live *I. sakaiensis* cells and the substrate PET or EG are required for the anaerobic conversion of PET or EG to acetate and ethanol (Table S4). ^1^H and ^13^C NMR spectroscopy of experiments with ^13^C labelled EG as the substrate (Figure 2C, D) and comparison with commercially available ^13^C EG, ^13^C ethanol, and ^13^C acetate samples (Figure S4, S5) confirmed that the products are derived from EG. A ^12^C acetate peak is also observed, which is consistent with the use of internal carbon storage compounds before consumption of the ^13^C labelled substrate EG.

Based on the NMR spectroscopy data and supported by genome analysis, we propose that under anaerobic conditions, EG is first dehydrated to acetaldehyde by ethanolamine ammonia lyase (EAL) and then converted by alcohol dehydrogenase (ADH) and aldehyde dehydrogenase (ALDH) to ethanol and acetyl‐CoA, respectively. The latter is further converted into acetate by phosphate acetyl transferase (PTA) and acetate kinase (ACK) coupled to ATP formation by substrate‐level phosphorylation (Figure 1). Both, acetate, and ethanol are transported outside of the cell. Ethanol can reenter the cell and be converted further into acetate (Figure 1). A similar anaerobic EG metabolism was observed in the acetogen *Acetobacterium woodii*.^[22]^ Genome analysis of *I. sakaiensis* supports our proposed EG metabolism under anaerobic conditions (Figure 1) as all mentioned enzymes (EAL, ADH, ALDH, PTA, and ACK) are present in the bacterial genome (Table S2).^[19]^ Dehydration of EG to acetaldehyde commonly employs diol dehydratase (*pdu* gene),^[22]^ which is unavailable in the genome of *I. sakaiensis*. However, its homolog EAL (*eut* gene) is available and previous studies have demonstrated that the *eut* bacterial microcompartment shares similar features with that of the *pdu* microcompartment in terms of encoded enzymes and chemical reactions.[^26^, ^27^, ^28^, ^29^] Further, it has been shown that *Salmonella enterica* can dehydrate diol to acetaldehyde when a *pdu* enzyme was replaced by the *eut* homolog.^[30]^ Previous observations therefore confirm that the *eut* gene in *I. sakaiensis* can dehydrate EG to acetaldehyde.

### Ethanol Fermentation

We noticed that during the EG metabolism, ethanol was further degraded into acetate when EG was almost consumed (Figure 2B). To investigate the possibility of ethanol conversion, we cultured *I. sakaiensis* directly with ^13^C labelled ethanol and ^1^H and ^13^C NMR spectroscopy confirmed the gradual fermentation of ethanol to acetate (Figure S6, S7). Based on this observation, we propose that the initially produced ethanol during EG and PET fermentation can reenter the cell, followed by conversion to acetaldehyde by ADH and further fermentation to acetate (Figure 1).

### Maltose Fermentation

It has previously been shown that *I. sakaiensis* can neither grow aerobically on glucose nor ferment it due to the lack of transporters for glucose uptake in *I. sakaiensis*, but aerobic growth with maltose has been verified previously.[^19^, ^21^] Our genome search suggested the possibility of maltose fermentation by *I. sakaiensis* (Table S2) and fermentative growth of *I. sakaiensis* was therefore also studied with maltose (40 mM) as the sole carbon source under anaerobic conditions. We detected the following fermentation products by ^1^H NMR spectroscopy: lactate, formate, acetate, and ethanol (Figure S8, S9). Under aerobic conditions, maltose is oxidized to CO_2_ and H_2_O through the TCA cycle (Figure S10). Under anaerobic conditions, maltose is fermented to a product pool via a protein network starting from the outer membrane to the cytoplasm in *I. sakaiensis* (Figure S10).[^31^, ^32^, ^33^] All the necessary proteins are available in the genome of *I. sakaiensis* with the exception of pyruvate formate lyase (PFL), which has yet to be identified (Table S2).^[19]^

### Co‐culturing of *I. sakaiensis* and *G. sulfurreducens* in a Bio‐electrochemical Cell

Bacteria can live in symbiosis, both in natural communities and artificial co‐culture systems. The co‐culturing promotes substrate utilization by serving the metabolites of one community to the neighboring community for their growth.[^34^, ^35^] Here we developed an artificial co‐culture system using *G. sulfurreducens* and *I. sakaiensis. G. sulfurreducens* usually lives with fermenting communities as they grow with acetate, one of the end products of fermentation.^[36]^ *G. sulfurreducens* is a gram‐negative, anaerobic, dissimilatory metal‐reducing bacterium with high electricity producing capacity in microbial fuel cells. *G. sulfurreducens* can transport metabolically generated electrons via acetate oxidation to a poised electrode through an extracellular electron transfer (EET) respiratory pathway.^[37]^ They also produce conductive protein nanowires for transporting metabolically generated electrons to insoluble electron acceptors such as metal oxide and metal electrodes.^[38]^

**Figure 3:**
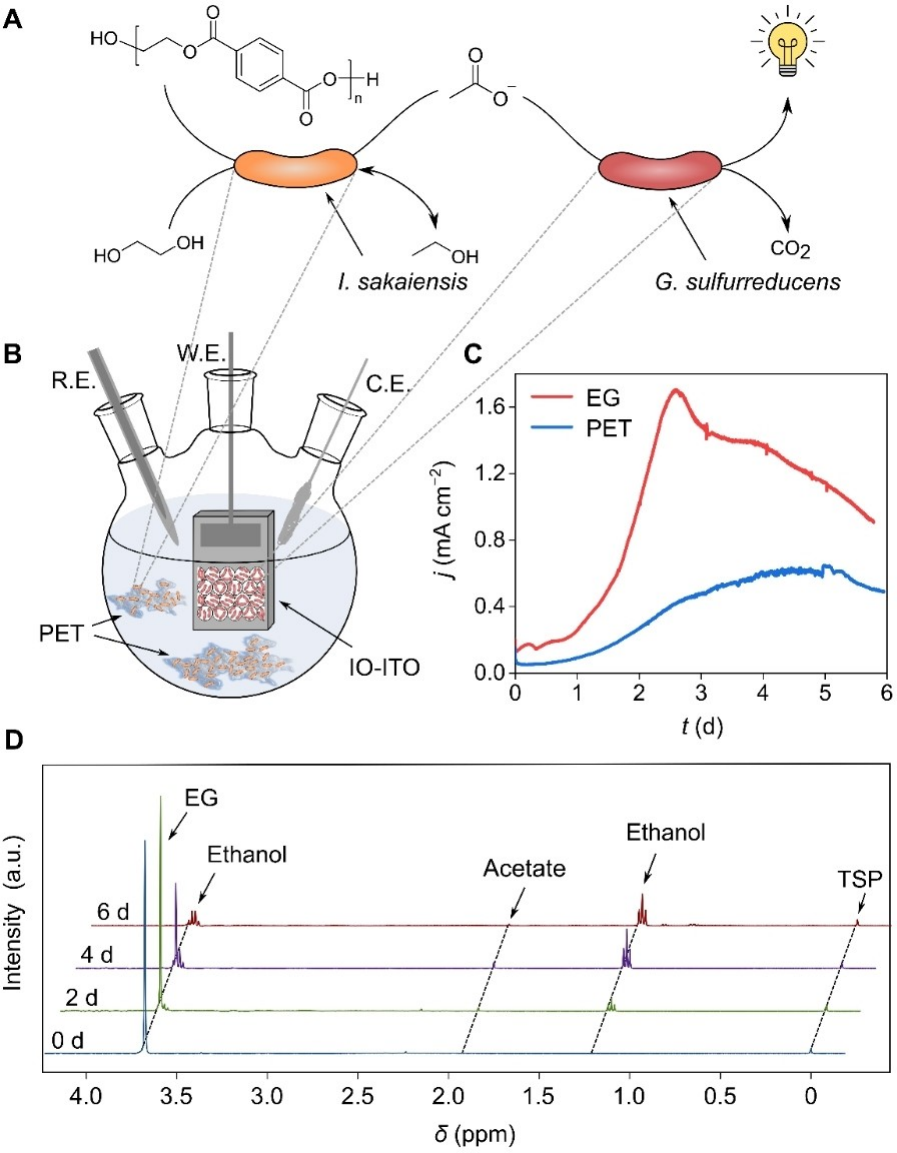
Electricity generation from PET and EG by a co‐culture of *I. sakaiensis* and immobilized *G. sulfurreducens* in a three‐electrode bio‐electrochemical system. A) PET and EG conversion to electricity and CO_2_ via intermediate acetate production by a co‐culture of *I. sakaiensis* and *G. sulfurreducens*. B) Schematic representation of the co‐culturing experiment in a bio‐electrochemical cell with a three‐electrode system. C) Conditions: *I. sakaiensis* OD_600_=1.2–1.4, IO‐ITO|*G. sulfurreducens* working electrode (W. E.) (Figure S11), 60 mg PET (blue) or 25 mM EG (red), 15 mL bicarbonate‐buffered medium, N_2_‐CO_2_ (80–20 %), 30 °C, 400 rpm, pH 7, 0.10 V vs. SHE, Ag/AgCl reference electrode (R.E.), Pt mesh counter electrode (C.E.). D) ^1^H NMR spectra before the start, after 2 d, 4 d, and 6 d of the chronoamperometry with 25 mM EG. The TSP signal indicates 0 ppm in each spectrum. The spectra are vertically and horizontally shifted as indicated by the black dashed lines.

We co‐cultured *I. sakaiensis* with *G. sulfurreducens* in a bio‐electrochemical reactor, where *I. sakaiensis* supplies acetate from fermentation of PET or EG to *G. sulfurreducens* for electricity production (Figure 3A). The bio‐electrochemical reactor consisted of a three‐electrode system with a Ag/AgCl reference electrode, a Pt mesh counter electrode, and an IO‐ITO working electrode (Figure 3B). The IO‐ITO electrode served as a host structure for the *G. sulfurreducens* biofilm and had a geometrical surface area of 0.25 cm^2^, a film thickness of 40–45 μm, and a macropore size of 8–10 μm.[^39^, ^40^] In the first step towards establishing the artificial co‐culture, we grew an electrochemically active *G. sulfurreducens* biofilm on the IO‐ITO electrode (IO‐ITO|*G. sulfurreducens*) following a previously published procedure^[40]^ using *G. sulfurreducens* as the inoculum (OD_600_=0.6) and acetate (20 mM) as the sole carbon source in bicarbonate‐buffered medium by poising an applied potential of 0.1 V vs. standard hydrogen electrode (SHE). A current plateau at 1.7 mA cm^−2^ was observed after 3 d (Figure S11), indicating that *G. sulfurreducens* has colonized on the electrode while metabolizing acetate to CO_2_.^[40]^ In the second step, the bicarbonate‐buffered medium was replenished with fresh medium (without acetate and planktonic *G. sulfurreducens*) and then *I. sakaiensis* was added (OD_600_=1.2–1.4) together with PET films (60 mg) or EG (25 mM) as the sole substrate to the bio‐electrochemical reactor containing the IO‐ITO|*G. sulfurreducens* electrode.

The bio‐electrochemical system with PET as the sole substrate consumed 23 mg PET films in 6 d and showed a maximum current density of 0.6 mA cm^−2^, while EG was entirely consumed and showed a current density of 1.7 mA cm^−2^ (Figure 3C). The lower current from PET compared to EG is attributed to the slower hydrolysis step for the insoluble synthetic polymer. Pairing this co‐culture system for PET conversion with an O_2_‐reducing cathode in a two‐electrode configuration would provide an estimated voltage of approximately 0.4 V with the observed current density of 0.6 mA cm^−2^. This would correspond to a power density for the microbial fuel cell of approximately 0.2–0.3 mW cm^−2^.^[41]^

Current production started to decay after an initial peak current for both PET and EG (Figure 3C), which is commonly observed in microbial fuel cells and an indication for a limitation in acetate supply.^[42]^ *I. sakaiensis* was unable to produce current from EG in the absence of *G. sulfurreducens*, which implies non‐electric behavior of *I. sakaiensis* (Figure S12). Co‐culturing in the absence of PET and EG did not show any electricity production indicating that the oxidation of PET or EG was the source of the observed electricity production (Figure S13). ^1^H NMR analysis of the EG experiment (Figure 3D) shows a small increase in acetate over time, which reaches 0.8 mM after 4 d at its highest and then decreases to 0.3 mM after 6 d. For ethanol an increase over time is observed and the concentration is 8.0 mM after 6 d, while EG is completely consumed at this point. The detection of very small amounts of acetate indicates that the acetate produced by *I. sakaiensis* is instantly consumed by *G. sulfurreducens* for electricity production. At the same time, ethanol is initially accumulated (Figure 3D) as *G. sulfurreducens* cannot use ethanol as the carbon source (Figure S14). However, ethanol can be further converted into acetate by *I. sakaiensis* once EG is fully consumed as observed in the fermentation experiment with the monoculture (Figure 2B). Cross‐sectional scanning electron microscopy (SEM) confirmed that bacteria are inside the pores and on top of the IO‐ITO electrode (Figure S15).

The experiments show that *I. sakaiensis* ferments PET or EG to acetate and ethanol while *G. sulfurreducens* uses the fermented acetate as the substrate for electricity production while releasing CO_2_. Overall, the symbiotic system shows a conversion of 16.7 mM EG after 6 d via acetate and ethanol (0.3 mM and 8.0 mM still present in solution after 6 d) (Figure 3D) into electricity and CO_2_ and also serves as the carbon source for cell growth of *I. sakaiensis* and *G. sulfurreducens*. In contrast, the monoculture only showed a conversion of 9.7 mM after 6 d (Figure 2B). This 1.7‐fold increase in the EG consumption in the co‐culture suggests that the metabolism of EG by *I. sakaiensis* was enhanced when the strain was grown together with *G. sulfurreducens*, where acetate is constantly being consumed. A classic example for the enhanced metabolism in a co‐culture system is nitrification. In this syntropic process, ammonium‐oxidizing bacteria (AOB) oxidize ammonia to nitrite while nitrite‐oxidizing bacteria convert nitrite further to nitrate. This concert operation has been shown to accelerate the performance of AOB.^[34]^

## Conclusion

*I. sakaiensis* has been known to degrade PET to CO_2_ under aerobic conditions, and we demonstrate here an anaerobic metabolic pathway (30 °C, pH 7, 1 atm) to convert PET into acetate and ethanol. This new plastic fermentation process by *I. sakaiensis* is supported by isotopic labelling studies and genome analysis. The present results confirm that *I. sakaiensis* is a facultative anaerobe, which shows versatile metabolic pathways for PET consumption and utilization under anaerobic and aerobic conditions. Plastic contaminated areas, in particular landfills, may have varying oxygen levels as plastic materials prevent oxygen penetration from the atmosphere, which might make the ability for anaerobic plastic degradation a necessity for the bacteria populating these areas. This study identifies plastic fermentation as a potentially sustainable approach to combat plastic pollution and produce high‐value chemicals from waste through microbial degradation and biosynthesis. Additionally, the symbiotic association of *I. sakaiensis* with *G. sulfurreducens* paves a unique pathway to produce electricity from PET plastic waste. This work therefore reports a PET upcycling process, termed ‘plastic fermentation’, which has the potential to emerge as a technology that combines plastic waste mitigation with the production of value‐added chemicals or energy.

## Conflict of interest

The authors declare no conflict of interest.

1

## Supporting information

As a service to our authors and readers, this journal provides supporting information supplied by the authors. Such materials are peer reviewed and may be re‐organized for online delivery, but are not copy‐edited or typeset. Technical support issues arising from supporting information (other than missing files) should be addressed to the authors.

Supporting Information

## Data Availability

The raw data related to this article are available at the University of Cambridge data repository: https://doi.org/10.17863/CAM.83752.
