# Supplementary material for: Microbial Fermentation of Polyethylene Terephthalate (PET) Plastic Waste for the Production of Chemicals or Electricity
Source: Angew Chem Int Ed Engl. 2022 Oct 10;61(45):e202211057. doi: 10.1002/anie.202211057 (PMC9828132; doi:10.1002/anie.202211057)
Supplement: Supplementary file 1 — Supporting Information [file ANIE-61-0-s001.pdf]

Angewandte Chemie
Eine Zeitschrift der Gesellschaft Deutscher Chemiker

Supporting Information

# Microbial Fermentation of Polyethylene Terephthalate (PET) Plastic Waste for the Production of Chemicals or Electricity

*S. Kalathil, M. Miller, E. Reisner**

## Table of Contents

Materials and Methods....................................................2
Supporting Figures S1 to S15....................................................4
Supporting Tables S1 to S4....................................................13
Supporting References....................................................16

## Materials and Methods

Chemicals. The following chemicals and materials were obtained from commercial suppliers and used without further purification unless otherwise stated: 3-(trimethylsilyl)propionic-2,2,3,3-$d_4$ acid sodium salt (TSP, Sigma Aldrich, 98 atom % D), acetate-$^{13}C_2$ (Sigma Aldrich, 99 atom % $^{13}C$), deuterium oxide (Sigma Aldrich, 99.9 atom % D), ethanol (VWR Chemicals, absolute), ethanol-$^{13}C_2$ (Sigma Aldrich, 99 atom % $^{13}C$), ethylene glycol (EG, Breckland Scientific), ethylene glycol-$^{13}C_2$ (Sigma Aldrich, 99 atom % $^{13}C$), glutaraldehyde solution (grade I, 25% in $H_2O$, specifically purified for use a s an electron microscopy fixative), indium tin oxide (Sigma Aldrich, <50 nm particle size), indium tin oxide glass for electrodes (Sigma Aldrich, conductivity 8-12 Ω $sq^{-1}$), maltose (Fisher Chemical, general purpose grade), $N_2$ gas and $N_2$-$CO_2$ (80-20%) gas mixture (BOC Ltd.), osmium tetroxide solution (Sigma Aldrich, 2% in $H_2O$,), polyethylene terephthalate (PET, films derived from a plastic water bottle (still Scottish mountain water) purchased from Marks and Spencer Simply Food (Station Road, Cambridge CB1 2JW, UK), dried and ground using a coffee grinder into pieces ≤ 1 $cm^2$), polystyrene latex microsphere (PS beads, Alfa Aesar, 2.5 wt% dispersion in $H_2O$, diameter 10.0 µm), sodium acetate (Alfa Aesar, anhydrous, 99%), sodium fumarate (Sigma Aldrich, ≥99%). The information for all chemicals used for bacteria medium preparation can be found in Table S1 and S3.

Nuclear magnetic resonance (NMR) spectroscopy. $^1H$ and $^{13}C$ NMR spectra were recorded on a Bruker Neo Prodigy 400 MHz spectrometer (equipped with a prodigy BBO cryoprobe) in a mixture of $H_2O$-$D_2O$ (95-5%) and a Bruker Avance III 500 MHz spectrometer (equipped with a BBO smart probe) in a mixture of $H_2O$-$D_2O$ (95-5%) using $H_2O$ suppression by pre-saturation at room temperature. All samples were filtered with syringe filters (Millex-GP, pore size 0.22 µm, Merck) prior to analysis. 570 µL of the filtrate was added to 30 µL of 5 mM TSP in $D_2O$ to give 600 µL sample for NMR analysis. Chemical shifts are referenced relative to the protium / $^1H$ signal of TSP: δ = 0.00 ppm and TSP was used as internal standard for the quantification of all liquid products. Quantification was executed with TopSpin 4.0.8. The representation of the NMR spectra was done using MestReNova 12.0.0 and the spectra were corrected using automated phase and baseline correction and additional manual baseline correction for the residual $H_2O$ peak in spectra recorded without $H_2O$ suppression.

Scanning electron microscopy (SEM). SEM was carried out on a TESCAN MIRA3 FEG-SEM. All samples were fixated by incubation in glutaraldehyde solution (2.5% in $H_2O$) for 30 min. After washing in bicarbonate-buffered medium the dried samples were further fixated by incubation in osmium tetroxide for a minimum of 4 h. Then the samples were dehydrated by incubation in a series of ethanol solutions with increasing ethanol concentration (50, 70, 90, 100%) for 30 min each. After drying, all samples were additionally sputtered with a 10 µm layer of Pt prior to the SEM analysis.

Culturing and handling of bacteria. *I. sakaiensis* (NBRC 110686) was purchased from NBRC Japan and cultured in 15 mL NBRC no. 802 broth (Table S1) overnight under aerobic conditions. *G. sulfurreducens* PCA (ATCC 51573) was purchased from DSMZ Germany and cultured in 15 mL bicarbonate-buffered medium (Table S3) for 3 days under anaerobic conditions. Sodium acetate (20 mM) and sodium fumarate (50 mM) were added to serve as the electron donor and acceptor, respectively during *G. sulfurreducens* growth. Anaerobic vials and electrochemical cells were always prepared by purging 15 mL bicarbonate-buffered medium with a gas mixture of $N_2$-$CO_2$ (80-20%) for 1 h before and 15 min after the inoculation. All inoculated serum vials (aerobic and anaerobic) were kept in a shaking incubator (INCU-Shake MIDI, SciQuip) at 30 °C and 300 rpm for the above specified times. The concentration of all bacteria suspensions after the growth period was determined by measuring the optical density at 600 nm ($OD_{600}$) using a UV–vis spectrometer (Varian Cary 50, Agilent Technologies). As-grown cells were centrifuged (Centrifuge 5804, Eppendorf) for 4 min at 7000 rpm and then resuspended in bicarbonate-buffered medium. This washing process was repeated three times and the resulting bacteria suspension was added to the anaerobic serum vials to obtain a final $OD_{600}$ = 1.2-1.4 for all experiments.

Fermentation by *I. sakaiensis.* Anaerobic serum vials were prepared as described above containing bicarbonate-buffered medium (15 mL) under a $N_2$-$CO_2$ (80-20%) atmosphere and either maltose (40 mM), PET film (60 mg), or EG (25 mM) was added as the fermenting substrate. After inoculation with *I. sakaiensis* the vials were kept in a shaking incubator at 30 °C and 300 rpm for up to one month. Aliquots (800 µL) were removed periodically using a syringe and prepared for NMR analysis as described above. After the experiment, the remaining PET films were washed with 70% ethanol and water and dried at 40 °C overnight before post-experiment quantification. Isotopic experiments were performed in the presence of $^{13}C$ labelled EG or ethanol. For experiments with killed bacteria (Table S4), *I. sakaiensis* was treated in an autoclave (Prestige Medical, Portable Autoclave Classic Media, 121 °C) before addition to the anaerobic serum vials. All experiments were performed in triplicate.

Co-culturing of *I. sakaiensis* and *G. sulfurreducens* in an electrochemical cell. Inverse opal-indium tin oxide (IO-ITO) electrodes (geometrical surface area: 0.25 $cm^2$, thickness: 40-45 µm, macropore size: 8-10 µm) were prepared by a previously published co-assembly method using ITO nanoparticles (< 50 nm particle size) and 10.0 µm polystyrene beads and annealing at 500 °C for 20 min with a heating rate of 1 °C $min^{-1}$ (1). Co-culturing of *G. sulfurreducens* and *I. sakaiensis* was conducted in an anaerobic electrochemical cell with a three-electrode system consisting of an IO-ITO working electrode, a platinum mesh counter electrode, and a Ag/AgCl (in 3 M NaCl solution, + 0.20 V *vs*. SHE) reference electrode.

In the first step, a *G. sulfurreducens* biofilm was grown on the IO-ITO electrode following previously published procedures (1, 2). In short, *G. sulfurreducens* ($OD_{600}$ = 0.6) was added to an anaerobic electrochemical cell with bicarbonate-buffered medium (15 mL) and sodium acetate (20 mM) as the sole electron donor at a pH of 7 under a $N_2$-$CO_2$ (80-20%) atmosphere at 30 °C, and stirring at 400 rpm. The working electrode was poised at 0.10 V *vs.* SHE with a potentiostat (MultiEmStat3+). After obtaining a stable current

(Figure S11), the medium was replenished by a fresh bicarbonate-buffered medium (15 mL, without acetate and planktonic *G. sulfurreducens*). In the second step, as-grown *I. sakaiensis* ($OD_{600}$ = 1.2-1.4) was added as the co-culture together with either PET (60 mg) or EG (25 mM) as the sole electron donor. The co-culture was kept for several days at a pH of 7 under a $N_2$-$CO_2$ (80-20%) atmosphere at 30 °C, stirring at 400 rpm, and the working electrode was again poised at a potential of 0.1 V *vs*. SHE for several days. Aliquots (800 µL) were removed periodically using a syringe and prepared for NMR analysis as described above. Remaining PET films were quantified after the experiment as described above.

## Supporting Figures

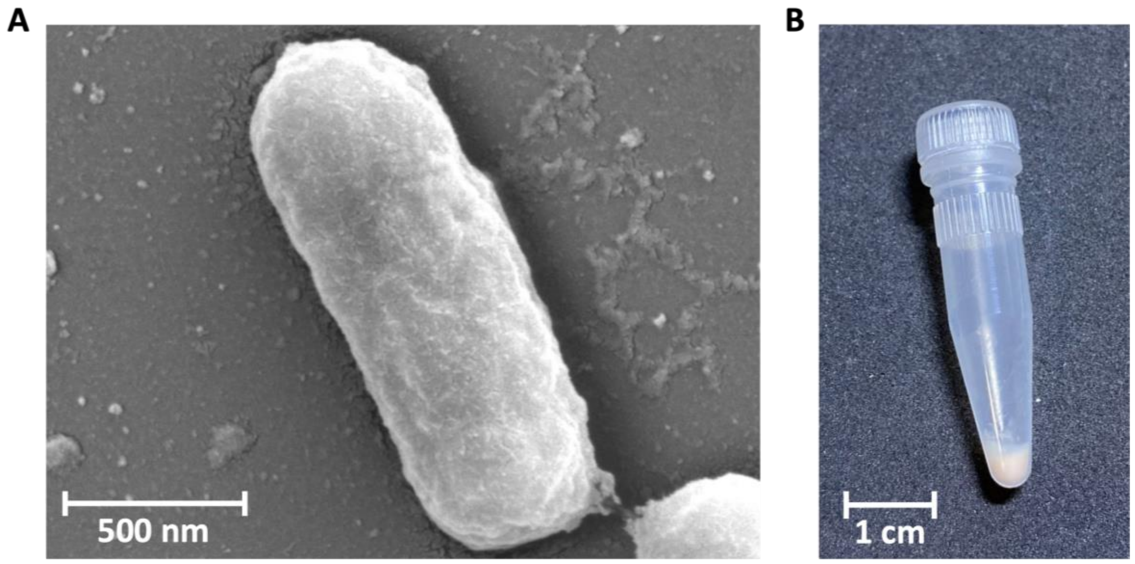


**Figure S1. Appearance of *I. sakaiensis*.** (A) SEM image of *I. sakaiensis* drop-casted on a flat ITO-coated glass substrate and (B) picture of an *I. sakaiensis* pellet after 24 h growth in NBRC no. 802 broth, washing and centrifugation in bicarbonate-buffered medium (3 x 10 mL).

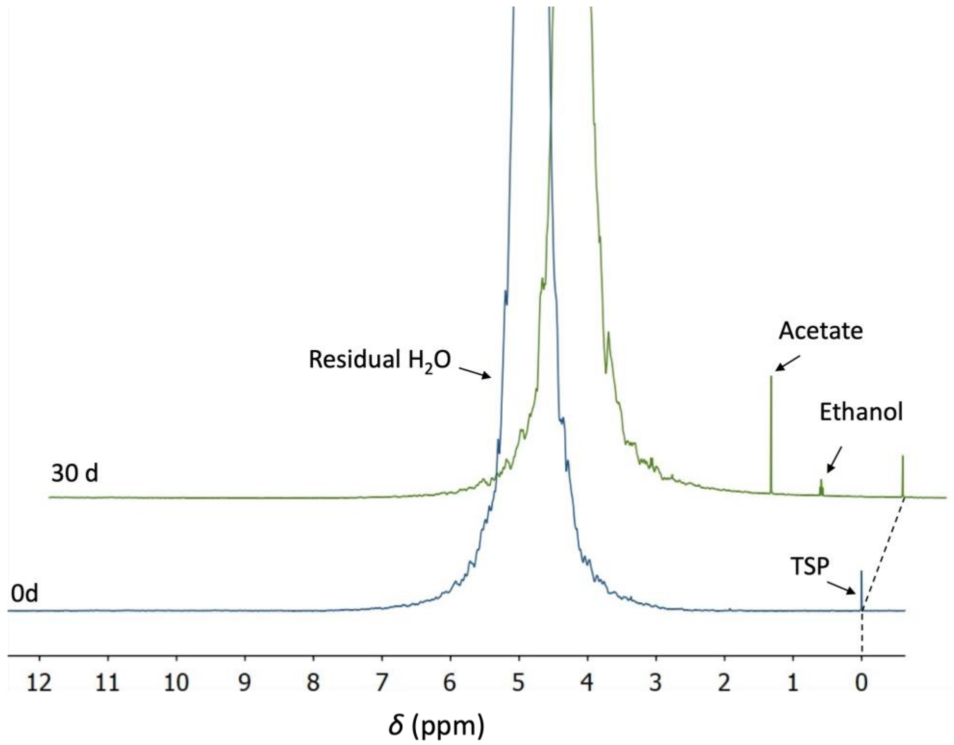


**Figure S2. $^1$H NMR spectra of anaerobic PET fermentation by *I. sakaiensis* over time.** Conditions: *I. sakaiensis* $OD_{600}$ = 1.2-1.4, 60 mg PET, 15 mL bicarbonate-buffered medium, $N_2$-$CO_2$ (80-20%), shaking incubator, 300 rpm, 30 °C, pH 7. The TSP signal indicates 0.0 ppm in each spectrum. The spectra are vertically and horizontally (black dashed line) shifted.

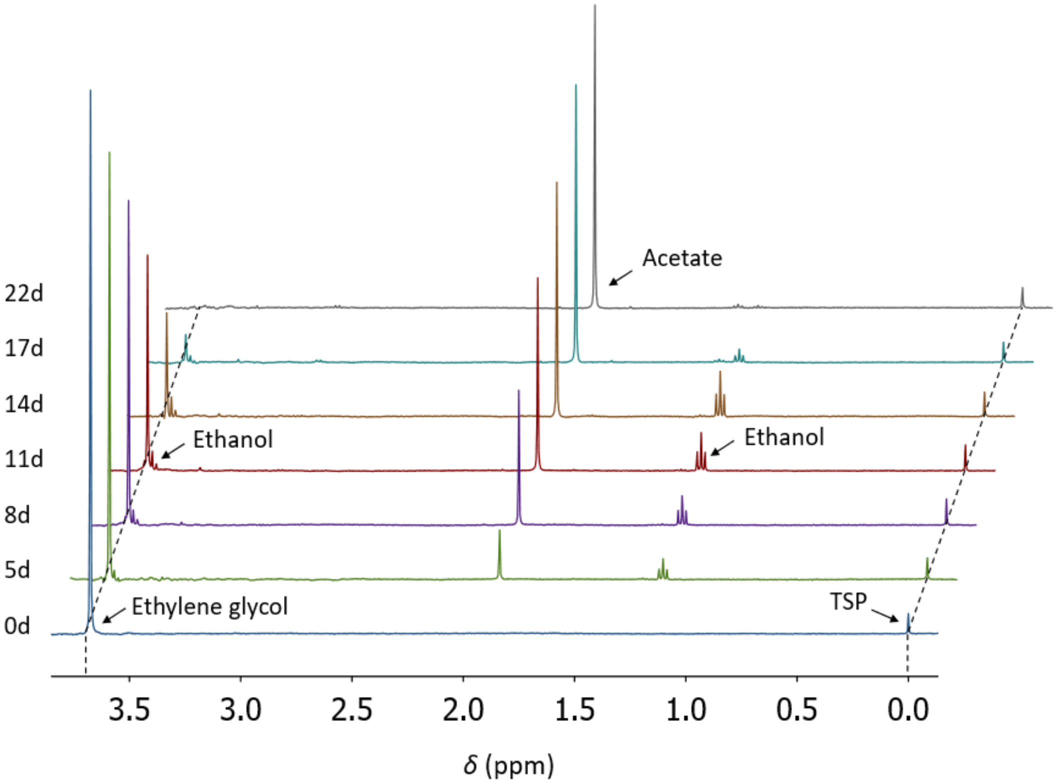


**Figure S3. $^{1}$H NMR spectra of anaerobic EG fermentation by *I. sakaiensis* over time.** Conditions: *I. sakaiensis* $OD_{600}$ = 1.2-1.4, 25 mM EG, 15 mL bicarbonate-buffered medium, $N_2$-$CO_2$ (80-20%), shaking incubator, 300 rpm, 30 °C, pH 7. The TSP signal indicates 0.0 ppm in each spectrum. The spectra are vertically and horizontally (black dashed line) shifted.

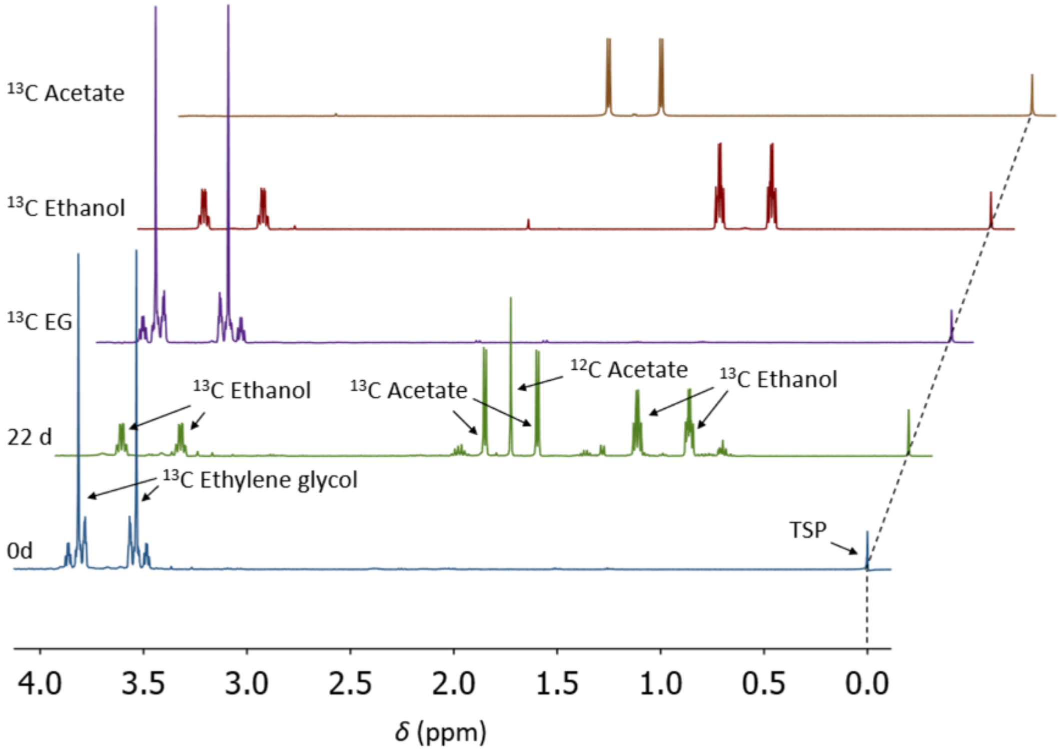


**Figure S4. $^{1}$H NMR spectra of anaerobic $^{13}$C EG fermentation by *I. sakaiensis* over time.** Conditions: *I. sakaiensis* $OD_{600}$ = 1.2-1.4, 25 mM $^{13}$C EG, 15 mL bicarbonate-buffered medium, $N_2$-$CO_2$ (80-20%), shaking incubator, 300 rpm, 30 °C, pH 7. A comparison with commercial samples of $^{13}$C EG, $^{13}$C ethanol, and $^{13}$C acetate is shown. The TSP signal indicates 0.0 ppm in each spectrum. The spectra are vertically and horizontally (black dashed line) shifted. The additional signals at 3.48, 3.56, 3.78, and 3.86 ppm in the 0 d NMR (blue) are assigned to impurities in the commercial sample of $^{13}$C EG (purple). These signals are not observed anymore after 22 d of reaction (green), where additional and unidentified signals are observed at 0.90, 1.48, 1.56, and 2.17 ppm instead. We believe that these signals are the result of the degradation of the impurities in the commercial sample of $^{13}$C EG as these signals are not observed in the experiment with $^{12}$C EG (Figure S3).

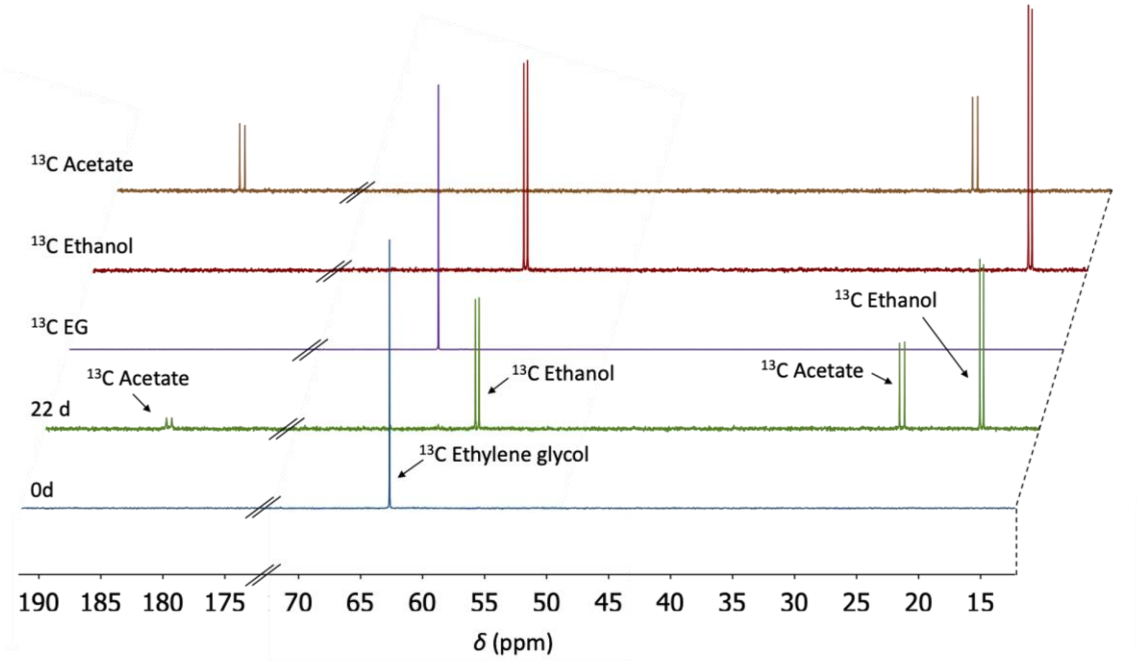


**Figure S5. $^{13}C$ NMR spectra of anaerobic $^{13}C$ EG fermentation by *I. sakaiensis* over time.** Conditions: *I. sakaiensis* $OD_{600}$ = 1.2-1.4, 25 mM $^{13}C$ EG, 15 mL bicarbonate-buffered medium, $N_2$-$CO_2$ (80-20%), shaking incubator, 300 rpm, 30 °C, pH 7. A comparison with commercial samples of $^{13}C$ EG, $^{13}C$ ethanol, and $^{13}C$ acetate is shown. The spectra are vertically and horizontally (black dashed line) shifted.

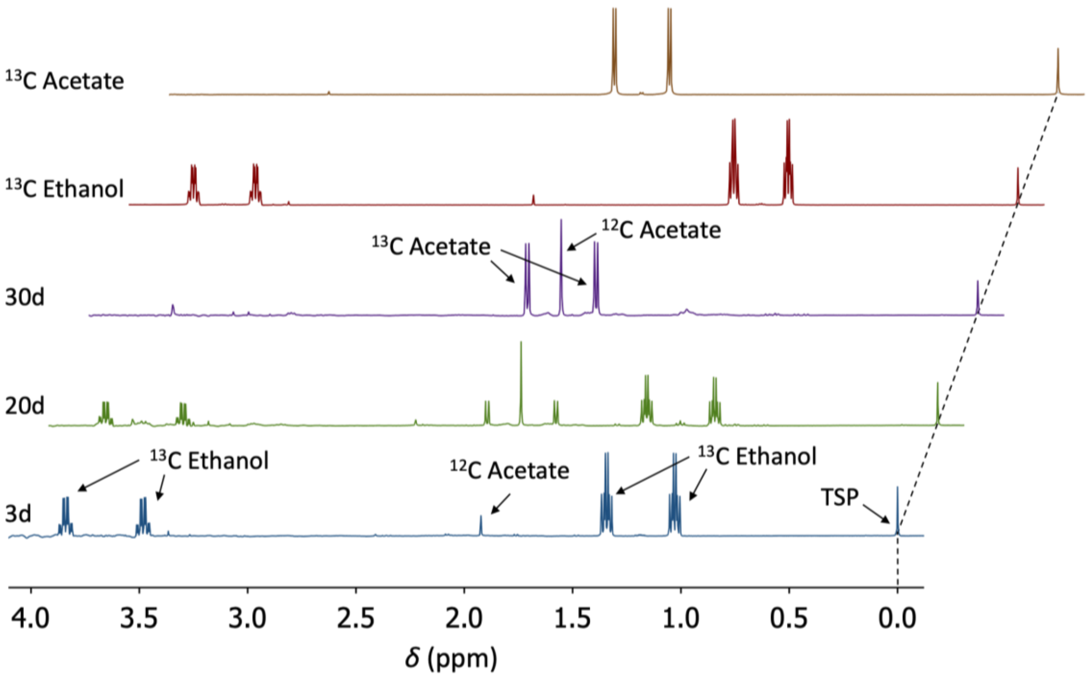


**Figure S6. $^{1}H$ NMR spectra of anaerobic $^{13}C$ ethanol fermentation by *I. sakaiensis* over time.** Conditions: *I. sakaiensis* $OD_{600}$ = 1.2-1.4, 25 mM $^{13}C$ ethanol, 15 mL bicarbonate-buffered medium, $N_2$-$CO_2$ (80-20%), shaking incubator, 300 rpm, 30 °C, pH 7. A comparison with commercial samples of $^{13}C$ ethanol and $^{13}C$ acetate is shown. The TSP signal indicates 0.0 ppm in each spectrum. The spectra are vertically and horizontally (black dashed line) shifted.

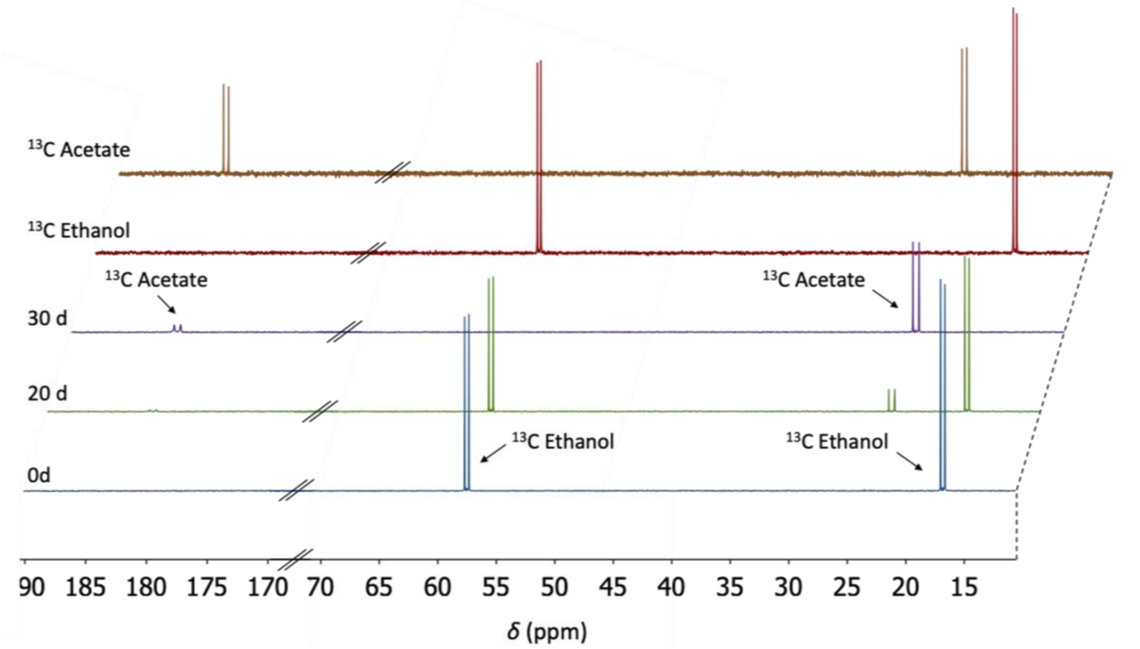


**Figure S7. $^{13}C$ NMR spectra of anaerobic $^{13}C$ ethanol fermentation by *I. sakaiensis* over time.** Conditions: *I. sakaiensis* $OD_{600}$ = 1.2-1.4, 25 mM $^{13}C$ ethanol, 15 mL bicarbonate-buffered medium, $N_2$-$CO_2$ (80-20%), shaking incubator, 300 rpm, 30 °C, pH 7. A comparison with commercial samples of $^{13}C$ ethanol and $^{13}C$ acetate is shown. The spectra are vertically and horizontally (black dashed line) shifted.

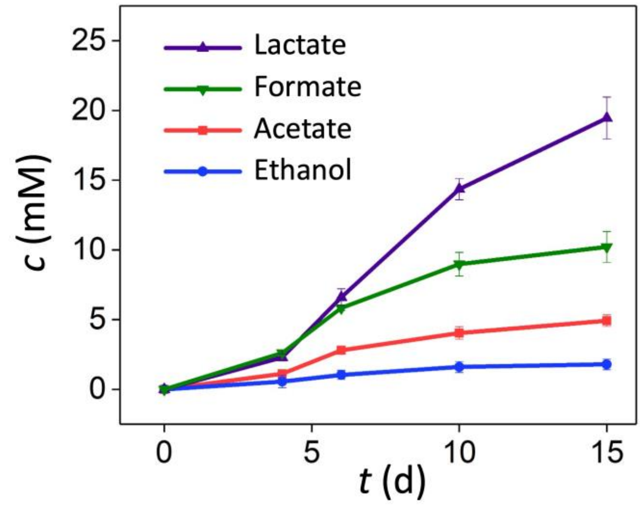


**Figure S8. Anaerobic maltose fermentation by *I. sakaiensis* over time.** Conditions: *I. sakaiensis* $OD_{600}$ = 1.2-1.4, 40 mM maltose, 15 mL bicarbonate-buffered medium, $N_2$-$CO_2$ (80-20%), shaking incubator, 300 rpm, 30 °C, pH 7. Error bars correspond to standard deviation (N = 3).

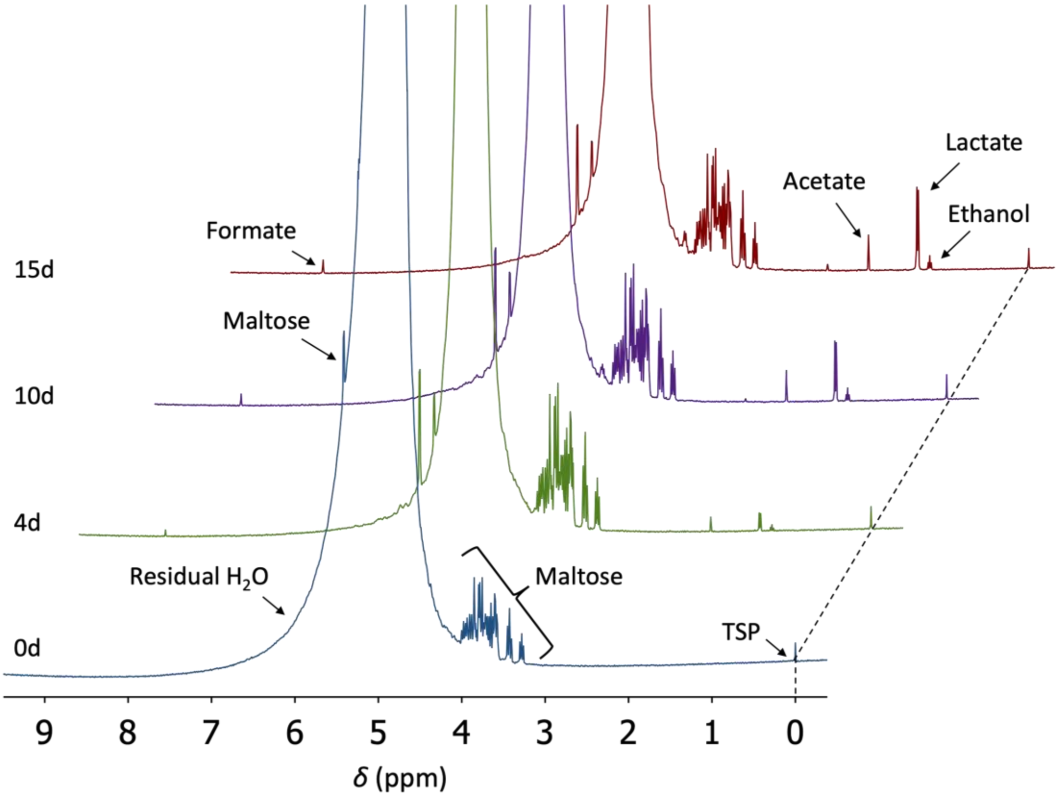


**Figure S9. $^1$H NMR spectra of the maltose fermentation by *I. sakaiensis* over time.** Conditions: *I. sakaiensis* $OD_{600}$ = 1.2-1.4, 40 mM maltose, 15 mL bicarbonate-buffered medium, $N_2$-$CO_2$ (80-20%), shaking incubator, 300 rpm, 30 °C, pH 7. The TSP signal indicates 0.0 ppm in each spectrum. The spectra are vertically and horizontally (black dashed line) shifted.

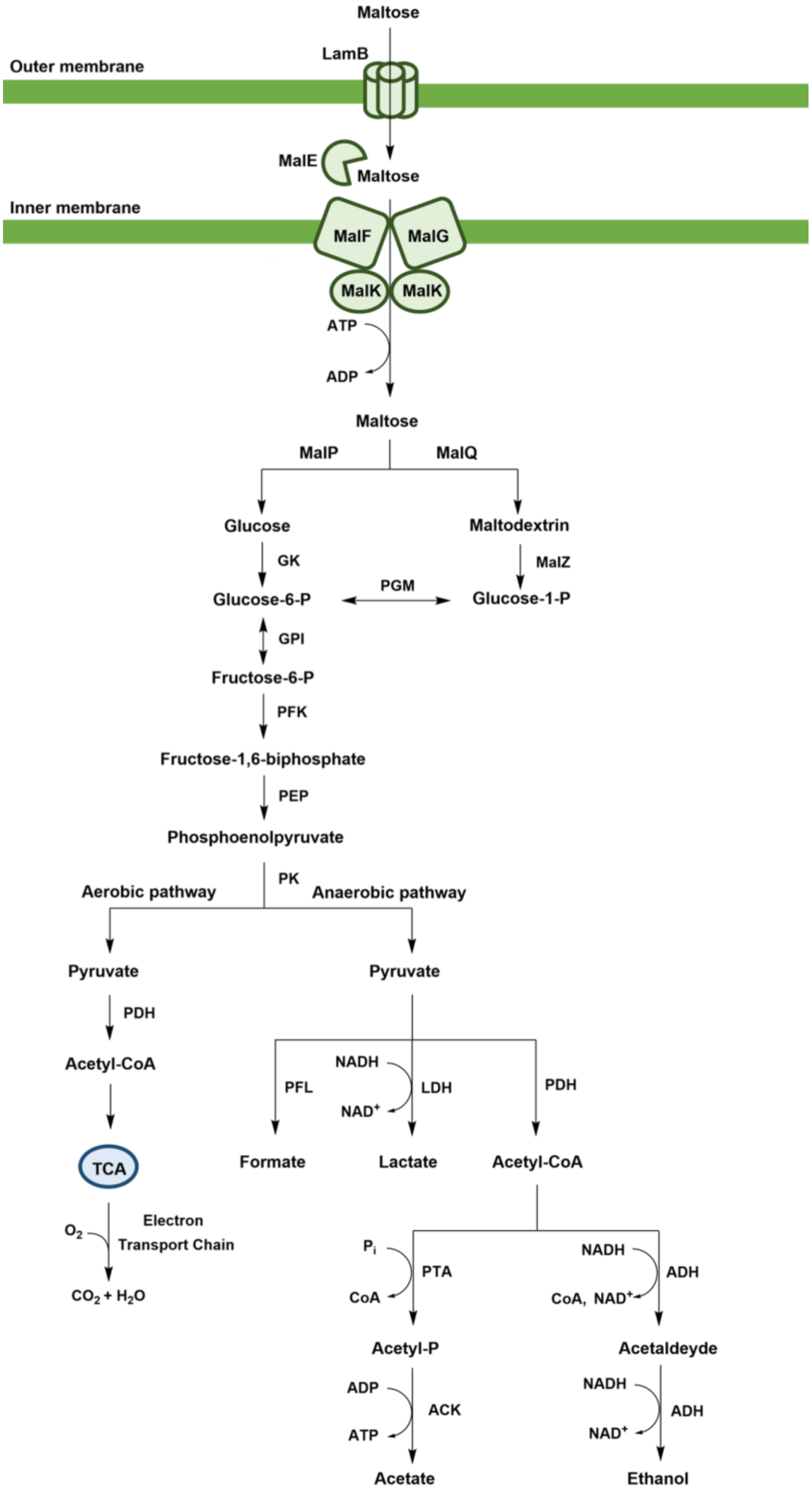


**Figure S10. Key proteins involved in the maltose fermentation.** Maltose was initially diffused through maltoporin (LamB) and taken up by the ABC transporters, substrate binding periplasmic protein MalE, permease proteins MalF and MalG, and ATP binding proteins MalK. Incoming maltose was metabolized to glucose and maltodextrin by a cytoplasmic enzyme, amylomaltase (MalQ). The glucose and maltodextrin were converted into glucose-6-P and glucose-1-P by glucose kinase (GK) and maltodextrin glucosidase (MalZ), respectively. Glucose-1-P was further converted to glucose-6-P by the action of phosphoglucomutase (PGM). Glucose-6-phosphate isomerase (GPI) converted glucose-6-P to fructose-6-P which further turned to fructose-1,6 biphosphate by phosphofructokinase (PFK). Fructose-1,6 biphosphate was degraded to phosphoenolpyruvate by phosphoenolpyruvate synthase (PEP) which was converted to pyruvate by pyruvate kinase (PK). Pyruvate was converted to acetyl-CoA by pyruvate dehydrogenase (PDH). In the anaerobic pathway, acetyl-CoA was converted to lactic and formic acids by the action of lactate dehydrogenase (LDH) and pyruvate formate lyase (PFL), respectively. Acetyl-CoA was also converted to ethanol by alcohol dehydrogenase (ADH) and to acetate by the combined actions of phosphate acetyltransferase (PTA) and acetate kinase (ACK). In the aerobic pathway, acetyl-CoA enters the tricarboxylic acid cycle (TCA) and the electron transport chain to yield the end products $CO_2$ and $H_2O$ (3). All mentioned enzymes are present in the genome of *I. sakaiensis* (Table S2) with the exception of PFL, which has yet to be identified (4).

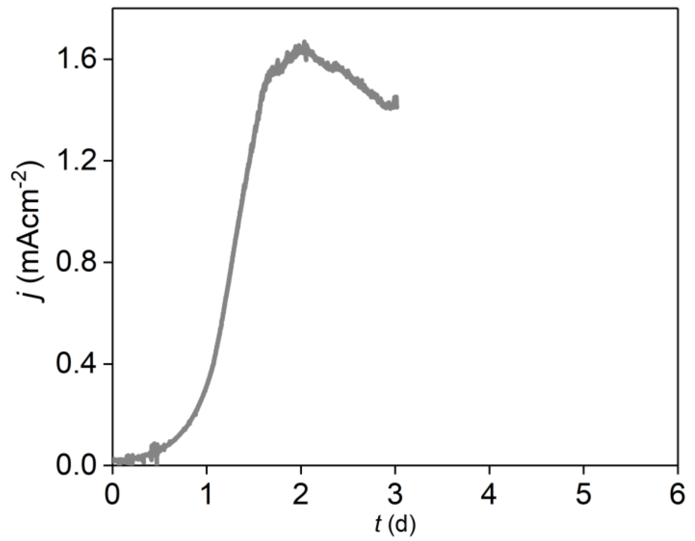


**Figure S11. Electricity generation by *G. sulfurreducens* from acetate in a three-electrode bio-electrochemical system over time.** A *G. sulfurreducens* biofilm was grown on an IO-ITO electrode following a previously published procedure (1). The current started to decay after 2 days due to the consumption of acetate. Conditions: *G. sulfurreducens* $OD_{600}$ = 0.6, 20 mM acetate, 15 mL bicarbonate-buffered medium, $N_2$-$CO_2$ (80-20%), 30 °C, 400 rpm, 0.1 V *vs.* SHE, IO-ITO working electrode, Ag/AgCl reference electrode, Pt mesh counter electrode.

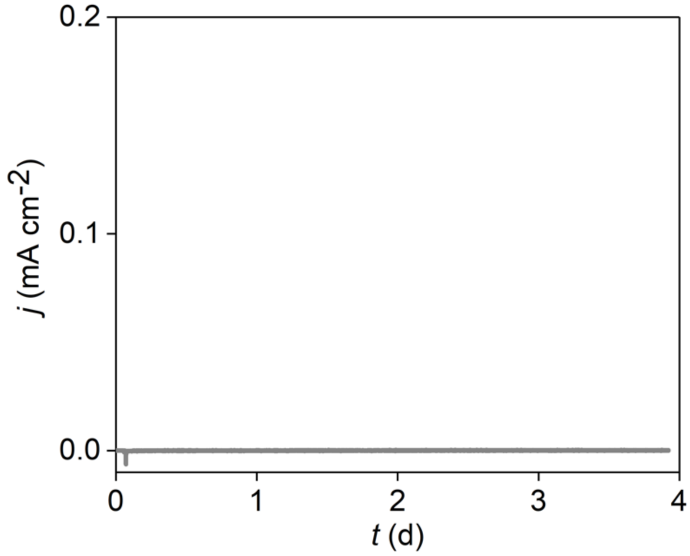


**Figure S12. Chronoamperometry of *I. sakaiensis* in the presence of EG in a three-electrode bio-electrochemical system over time.** Conditions: *I. sakaiensis* $OD_{600}$ = 1.2-1.4, 25 mM EG, 15 mL bicarbonate-buffered medium, $N_2$-$CO_2$ (80-20%), 30 °C, 400 rpm, pH 7, 0.1 V *vs.* SHE, IO-ITO working electrode, Ag/AgCl reference electrode, Pt mesh counter electrode.

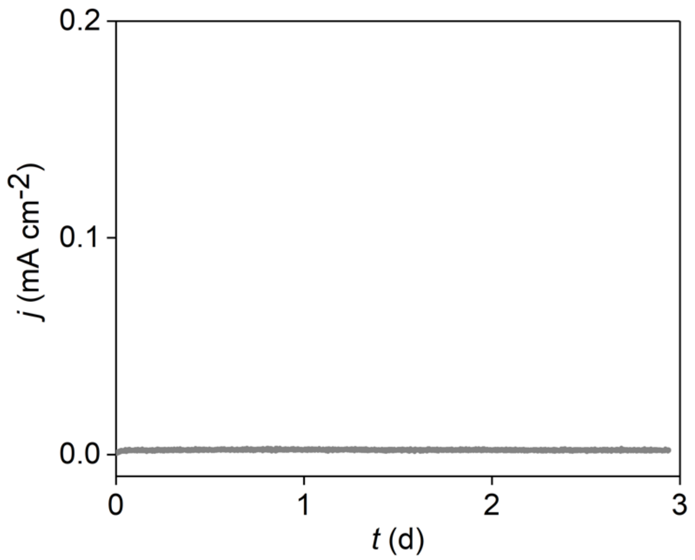


**Figure S13. Chronoamperometry of a co-culture of *G. sulfurreducens* and *I. sakaiensis* in the absence of PET and EG in a three-electrode bio-electrochemical system over time.** Conditions: *G. sulfurreducens* biofilm on an IO-ITO working electrode (Figure S11), *I. sakaiensis* $OD_{600}$ = 1.2-1.4, 15 mL bicarbonate-buffered medium, $N_2$-$CO_2$ (80-20%), 30 °C, 400 rpm, pH 7, 0.1 V *vs.* SHE, Ag/AgCl reference electrode, Pt mesh counter electrode.

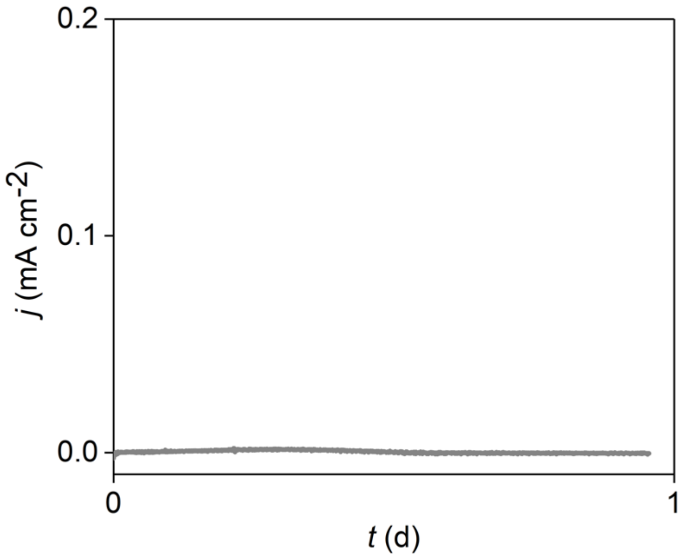


**Figure S14. Chronoamperometry of *G. sulfurreducens* in the presence of ethanol in a three-electrode bio-electrochemical system over time.** Conditions: *G. sulfurreducens* $OD_{600}$ = 0.6, 20 mM ethanol, 15 mL bicarbonate-buffered medium, $N_2$-$CO_2$ (80-20%), 30 °C, 400 rpm, pH 7, 0.1 V *vs.* SHE, IO-ITO working electrode, Ag/AgCl reference electrode, Pt mesh counter electrode.

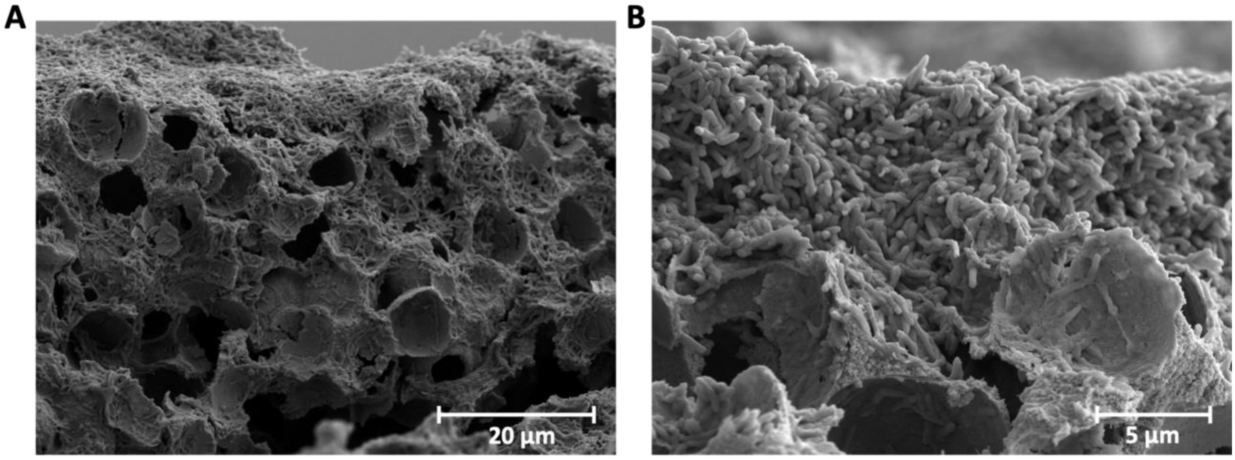


**Figure S15. Cross-section SEM images of an IO-ITO|*G. sulfurreducens* electrode after the co-culturing experiment with *I. sakaiensis* in a three-electrode bio-electrochemical system**.
(**A**) Full cross section and (**B**) top of an IO-ITO electrode with a thickness of 40-45 µm, a macropore size of 8-10 µm, and a pre-grown *G. sulfurreducens* biofilm (Figure S11) after the co-culturing experiment with *I. sakaiensis*.

## Supporting Tables

**Table S1. Components of NBRC no. 802 broth for aerobic culturing of *I. sakaiensis*.**

| | |
|---|---|
| Peptone | 10.00 g |
| Yeast extract | 2.00 g |
| $MgSO_4 \cdot 7 H_2O$ | 1.00 g |
| MilliQ water | 1000.00 mL |

**Table S2. Key anaerobic and fermenting enzymes present in the genome of *I. sakaiensis*.** The table shows key anaerobic and fermenting enzymes that generally indicate a facultative trait of *I. sakaiensis*, key enzymes for anaerobic PET and EG fermentation, and key enzymes for maltose fermentation. The table was adapted from the previously published genome (4).

| Key anaerobic and fermenting enzymes in the genome of *I. sakaiensis* | Gene name |
|---|---|
| Anaerobic dehydrogenases | ISF6_0313 |
| Anaerobic dimethyl sulfoxide reductase, chain A (EC 1.8.99.-) | ISF6_4180 |
| Anaerobic dimethyl sulfoxide reductase, chain C (EC 1.8.99.-) | ISF6_4182 |
| Assimilatory nitrate reductase, large subunit (EC 1.7.99.4) | ISF6_2606 |
| Carbon monoxide dehydrogenase D | ISF6_2776 |
| Carbon monoxide dehydrogenase G protein | ISF6_2774 |
| Carbon monoxide dehydrogenase, large chain (EC 1.2.99.2) | ISF6_3633 |
| Carbon monoxide dehydrogenase, medium chain (EC 1.2.99.2) | ISF6_3631 |
| Carbon monoxide dehydrogenase, small chain (EC 1.2.99.2) | ISF6_3632 |
| Carbon monoxide dehydrogenase, small chain (EC 1.2.99.2) | ISF6_2249 |
| Carbon monoxide dehydrogenase, small chain (EC 1.2.99.2) | ISF6_2779 |
| Cytochrome c biogenesis protein | ISF6_3455 |
| Cytochrome c domain-containing protein | ISF6_1712 |
| Cytochrome c family protein | SF6_3969 |
| Cytochrome c heme lyase, subunit CcmF | ISF6_2677 |
| Cytochrome c mono-and diheme variant | ISF6_1743 |
| Cytochrome c subunit of flavocytochrome c sulfide dehydrogenase | ISF6_1194 |
| Cytochrome c-type biogenesis protein CcmG/DsbE | ISF6_2678 |
| Cytochrome c-type biogenesis protein DsbD | ISF6_4634 |
| Di-heme cytochrome c (Class I) | ISF6_2005 |
| Ferredoxin subunit of nitrite reductase | ISF6_0047 |
| Ferric uptake regulation protein | fur ISF6_1293 |
| Fe-S oxidoreductase (EC 1.8.-.-) | ISF6_4919 |
| Formate dehydrogenase O, alpha subunit (EC 1.2.1.2) | ISF6_0980 |
| Formate dehydrogenase O, gamma subunit (EC 1.2.1.2) | ISF6_0984 |
| Formate dehydrogenase-O, major subunit (EC 1.2.1.2) | ISF6_5028 |
| Glucose-6-phosphate 1-dehydrogenase (G6PD) (EC 1.1.1.49) | zwf ISF6_3292 |
| Glucose-6-phosphate 1-dehydrogenase (G6PD) (EC 1.1.1.49) | zwf ISF6_0308 |
| Glyceraldehyde-3-phosphate dehydrogenase (EC 1.2.1.-) | ISF6_4649 |
| Heme exporter protein C (Cytochrome c-type biogenesis protein) | ccmC ISF6_2674 |
| High-potential iron-sulfur protein (HiPIP) | ISF6_4573 |
| Histidine kinase (EC 2.7.13.3) | ISF6_4663 |
| Histidine kinase (EC 2.7.13.3) | ISF6_1375 |
| Indole pyruvate ferredoxin oxidoreductase, alpha and beta subunits | ISF6_2075 |
| Iron-sulfur cluster carrier protein | ISF6_0978 |
| Isocitrate dehydrogenase [NADP] (EC 1.1.1.42) | ISF6_0156 |
| Malate dehydrogenase (EC 1.1.1.37) | mdh ISF6_2428 |
| NAD-dependent formate dehydrogenase, alpha subunit | ISF6_3288 |
| NAD-dependent formate dehydrogenase, beta subunit | ISF6_3287 |
| NAD-dependent formate dehydrogenase, delta subunit | ISF6_3290 |
| NAD-dependent formate dehydrogenase, gamma subunit | ISF6_3286 |
| [NiFe] hydrogenase metallocenter assembly protein HypC | ISF6_5487 |

| | | |
|---|---|---|
| [NiFe] hydrogenase metallocenter assembly protein HypE | | ISF6_5489 |
| [NiFe] hydrogenase metallocenter assembly protein HypF | | ISF6_5486 |
| [NiFe] hydrogenase nickel incorporation-associated protein HypB | | ISF6_5485 |
| Nitrite reductase [NADPH], large subunit (EC 1.7.1.4) | | ISF6_2608 |
| Nitrite reductase [NADPH], small subunit (EC 1.7.1.4) | | ISF6_2607 |
| Phosphoglycerate kinase (EC 2.7.2.3) | | pgk ISF6_3710 |
| Putative diheme cytochrome c-553 | | ISF6_2010 |
| Putative sulfite reductase | | ISF6_3405 |
| Succinate dehydrogenase flavoprotein subunit (EC 1.3.5.1) | | ISF6_2424 |
| Succinate dehydrogenase iron-sulfur subunit (EC 1.3.5.1) | | ISF6_2423 |
| Succinate dehydrogenase iron-sulfur subunit (EC 1.3.5.1) | | ISF6_3332 |
| Succinate dehydrogenase, cytochrome b subunit family protein | | ISF6_2003 |
| Succinate dehydrogenase, cytochrome b-556 subunit | | ISF6_2426 |
| Succinate dehydrogenase, cytochrome b-556 subunit | | ISF6_3334 |
| Succinate dehydrogenase, flavoprotein subunit | | ISF6_3338 |
| Succinate dehydrogenase, hydrophobic membrane anchor protein | | ISF6_2425 |
| Succinyl-CoA synthetase, alpha subunit-related enzyme | | ISF6_3279 |
| Sulfite reductase [NADPH] hemoprotein, beta-component (EC 1.8.1.2) | | ISF6_1463 |
| Transcriptional regulator, Crp/Fnr family | | ISF6_4716 |
| Transcriptional regulator, Crp/Fnr family | | ISF6_1659 |
| Transcriptional regulator, Crp/Fnr family | | ISF6_5057 |
| Transcriptional regulator, Crp/Fnr family | | ISF6_0152 |
| Transcriptional regulator, Crp/Fnr family | | ISF6_2369 |
| Transcriptional regulator, Crp/Fnr family | | ISF6_4999 |
| Ubiquinol-cytochrome c reductase iron-sulfur subunit (EC 7.1.1.8) | | ISF6_2599 |

| **Key enzymes for the anaerobic EG and PET fermentation present in the genome of *I. sakaiensis*** | **Abbreviation** | **Gene name** |
|---|---|---|
| Acetate kinase (EC 2.7.2.1) (Acetokinase) | ACK | ackA ISF6_3956 |
| Acetate kinase (EC 2.7.2.1) (Acetokinase) | ACK | ackA ISF6_4535 |
| Alcohol dehydrogenase (EC 1.1.1.1) | ADH | ISF6_4201 |
| Alcohol dehydrogenase (EC 1.1.1.1) | ADH | ISF6_1729 |
| Aldehyde dehydrogenase (EC 1.2.1.3) | ALDH | ISF6_4549 |
| Aldehyde dehydrogenase (EC 1.2.1.3) | ALDH | ISF6_4484 |
| Aldehyde dehydrogenase (EC 1.2.1.3) | ALDH | ISF6_2026 |
| Ethanolamine ammonia-lyase, heavy chain (EC 4.3.1.7) | EAL | ISF6_3350 |
| Ethanolamine ammonia-lyase light chain (EC 4.3.1.7 | EAL | ISF6_3349 |
| Phosphate acetyltransferase (EC 2.3.1.8) | PTA | ISF6_4168 |

| **Key enzymes for the anaerobic Maltose fermentation present in the genome of *I. sakaiensis*** | **Abbreviation** | **Gene name** |
|---|---|---|
| 4-alpha-glucanotransferase (EC 2.4.1.25) (Amylomaltase) (Disproportionating enzyme) | MalQ | ISF6_2253 |
| Acetate kinase (EC 2.7.2.1) (Acetokinase) | ACK | ackA ISF6_3956 |
| Acetate kinase (EC 2.7.2.1) (Acetokinase) | ACK | ackA ISF6_4535 |
| Acetyltransferase component of pyruvate dehydrogenase complex (EC 2.3.1.12) | PDH | ISF6_1479 |
| Alcohol dehydrogenase (EC 1.1.1.1) | ADH | ISF6_4201 |
| Alcohol dehydrogenase (EC 1.1.1.1) | ADH | ISF6_1729 |
| Dihydrolipoamide dehydrogenase of pyruvate dehydrogenase complex (EC 1.8.1.4) | PDH | ISF6_0563 |
| Glucokinase (EC 2.7.1.2) (Glucose kinase) | GK | glk ISF6_2896 |
| Glucose-6-phosphate isomerase (GPI) (EC 5.3.1.9) | GPI | pgi ISF6_4130 |
| L-lactate dehydrogenase (EC 1.1.2.3) | LDH | ISF6_1311 |
| L-lactate dehydrogenase (EC 1.1.2.3) | LDH | ISF6_2130 |
| L-lactate dehydrogenase (EC 1.1.2.3) | LDH | ISF6_0998 |
| Maltodextrin glucosidase (EC 3.2.1.20) | MalZ | ISF6_4138 |

| | | |
|---|---|---|
| Maltoporin | LamB | ISF6_4144 |
| Maltose/maltodextrin ABC transporter, permease protein MalF | MalF | ISF6_3761 |
| Maltose/maltodextrin ABC transporter, permease protein MalG | MalG | ISF6_3760 |
| Maltose/maltodextrin ABC transporter, substrate binding periplasmic protein MalE | MalE | ISF6_4141 |
| Maltose/maltodextrin transport ATP-binding protein MalK (EC 3.6.3.19)- | MalK | ISF6_4137 |
| Phosphate acetyltransferase (EC 2.3.1.8) | PTA | ISF6_4168 |
| Phosphoenolpyruvate synthase (EC 2.7.9.2) | PEP | ISF6_4185 |
| Phosphoenolpyruvate synthase (PEP synthase) (EC 2.7.9.2) | PEP | ISF6_4844 |
| Phosphofructokinase | PFK | ISF6_3856 |
| Phosphoglucomutase (EC 5.4.2.2) | PGM | ISF6_4131 |
| Pyruvate dehydrogenase E1 component (EC 1.2.4.1) | PDH | ISF6_1478 |
| Pyruvate dehydrogenase E1 component (EC 1.2.4.1) | PDH | ISF6_1222 |
| Pyruvate formate lyase | PFL | to be identified |
| Pyruvate kinase (EC 2.7.1.40) | PK | ISF6_3709 |

**Table S3. Components of the bicarbonate-buffered medium.** This medium was used for anaerobic culturing of *G. sulfurreducens*, the co-culturing experiments, and the anaerobic growth experiments with *I. sakaiensis*.

| | |
|---|---|
| $NH_4Cl$ (Sigma Aldrich, 99.998% trace metals basis) | 1.50 g |
| $Na_2HPO_4$ (Sigma Aldrich, 99.95% trace metals basis) | 0.60 g |
| KCl (Sigma Aldrich, ≥99 %) | 0.10 g |
| Trace element solution (see below) | 10.00 mL |
| $NaHCO_3$ (Fisher Scientific, 99.7-100.3%) | 2.50 g |
| Vitamin solution (see below) | 10.00 mL |
| Milli-Q water | 980.00 mL |
| **Trace element solution** | |
| Nitrilotriacetic acid (Sigma Aldrich, ≥99 %) | 1.50 g |
| $MgSO_4 \cdot 7\ H_2O$ (Sigma Aldrich, BioUltra, ≥99.5%) | 3.00 g |
| $MnSO_4 \cdot H_2O$ (Sigma Aldrich, ≥99 %) | 0.50 g |
| NaCl (Fisher Bioreagents, ≥99.0 %) | 1.00 g |
| $FeSO_4 \cdot 7\ H_2O$ (Sigma Aldrich, ≥99.0 %) | 0.10 g |
| $CoSO_4 \cdot 7\ H_2O$ (Sigma Aldrich, 99.998% trace metals basis) | 0.18 g |
| $CaCl_2 \cdot 2\ H_2O$ (Sigma Aldrich, ≥99%) | 0.10 g |
| $ZnSO_4 \cdot 7\ H_2O$ (Sigma Aldrich, ≥99%) | 0.18 g |
| $CuSO_4 \cdot 5\ H_2O$ (Sigma Aldrich, ≥98%) | 0.01 g |
| $KAl(SO_4)_2 \cdot 12\ H_2O$ (Sigma Aldrich, ≥98%) | 0.02 g |
| $H_3BO_3$ (Sigma Aldrich, ≥99.50%) | 0.01 g |
| $Na_2MoO_4 \cdot 2\ H_2O$ (Sigma Aldrich, ≥99%) | 0.01 g |
| $NiCl_2 \cdot 6\ H_2O$ (Sigma Aldrich, ≥97%) | 0.03 g |
| $Na_2SeO_3 \cdot 5\ H_2O$ (Sigma Aldrich, 98%) | 0.30 g |
| $Na_2WO_4 \cdot 2\ H_2O$ (Sigma Aldrich, ≥99%) | 0.40 g |
| Milli-Q water | 1000.00 mL |
| **Vitamin solution** | |
| Biotin (Sigma Aldrich, ≥99%) | 2.00 mg |
| Folic acid (Sigma Aldrich, ≥97%) | 2.00 mg |
| Pyridoxine-HCl (Sigma Aldrich, ≥98%) | 10.00 mg |
| Thiamine-HCl • 2 $H_2O$ (Sigma Aldrich, ≥99%) | 5.00 mg |
| Riboflavin (Fisher Scientific, Across Organics, 98%) | 5.00 mg |
| Nicotinic acid (Sigma Aldrich, ≥98%) | 5.00 mg |
| D-Ca-pantothenate (Sigma Aldrich, ≥98%) | 5.00 mg |

| | |
|---|---|
| Vitamin B12 (Sigma Aldrich, ≥98%) | 0.10 mg |
| p-Aminobenzoic acid (Sigma Aldrich, ≥99%) | 5.00 mg |
| Lipoic acid (Sigma Aldrich, ≥98%) | 5.00 mg |
| MilliQ water | 1000.00 mL |

**Table S4. Product formation after 30 d for the anaerobic PET and EG fermentation by *I. sakaiensis* including deletional control experiments.** Conditions: *I. sakaiensis* $OD_{600}$ = 1.2-1.4, 15 mL bicarbonate-buffered medium, $N_2$-$CO_2$ (80-20%), shaking incubator, 300 rpm, 30 °C, pH 7, 30 d. For experiments with killed bacteria, *I. sakaiensis* was autoclaved prior to addition. ○ indicates that the component was present in the experiment, – indicates that the component was absent in the experiment, and n.d. stands for not detected.

| **Components in the experiment** | | | **Products** | |
|---|---|---|---|---|
| *I. sakaiensis* | PET | EG | Acetate | Ethanol |
| ○ | ○ | – | 1.9 | 0.4 |
| ○ | – | ○ | 21.8* | n.d.* |
| ○ | – | – | 0.3 mM | n.d. |
| – | ○ | – | n.d. | n.d. |
| – | – | ○ | n.d. | n.d. |
| killed | ○ | – | n.d. | n.d. |
| killed | – | ○ | n.d. | n.d. |

*values after 22 d

## Supporting References


[1] X. Fang, S. Kalathil, G. Divitini, Q. Wang, E. Reisner, *Proc. Natl. Acad. Sci.* **117**, 5074–5080 (2020).

[2] B. E. Logan, R. Rossi, A. Ragab, P. E. Saikaly, *Nat. Rev. Microbiol.* **17**, 307–319 (2019).

[3] W. Boos, H. Shuman, *Microbiol. Mol. Biol. Rev.* **62**, 204–229 (1998).

[4] S. Yoshida, K. Hiraga, T. Takehana, I. Taniguchi, H. Yamaji, Y. Maeda, K. Toyohara, K. Miyamoto, Y. Kimura, K. Oda, *Science* **351**, 1196–1199 (2016).


## Author Contributions

SK, MM, and ER conceived the idea and designed the project. SK cultured bacteria, performed fermentation, isotopic experiments, coculturing and electricity production from plastics. SK and MM performed NMR analyses. MM recorded SEM images of electrodes and biofilms. SK, MM, and ER analyzed the data and discussed the results. SK, MM, and ER wrote the manuscript. ER supervised the project.

End of Supporting Information
